# Association between education level and depressive symptom trajectories in middle-aged and older Chinese adults: a nationally representative cohort study

**DOI:** 10.1186/s40359-026-04794-x

**Published:** 2026-05-19

**Authors:** Dehua Zhao, Xiaoqing Long, Jisheng Wang

**Affiliations:** Department of Clinical Pharmacy, The Third Hospital of Mianyang (Sichuan Mental Health Center), Mianyang, China

**Keywords:** Education level, Depressive symptoms, Trajectories, CHARLS, GBTM

## Abstract

**Background:**

Previous studies reported that education level was associated with depressive symptoms, but its impact on various long-term depressive symptom trajectories has not been assessed. This study aimed to explore the association between education level and depressive symptom trajectories in middle-aged and older Chinese individuals.

**Methods:**

We utilized data from the China Health and Retirement Longitudinal Study (CHARLS). Depressive symptoms across five waves from 2011 to 2020 were evaluated using the 10-item Center for Epidemiologic Studies Depression Scale (CESD-10). Group-based trajectory modeling (GBTM) was used to identify diverse patterns of depressive symptom trajectories. Multivariable logistic regressions were conducted to assess the association between education level and depressive symptom trajectories. Additionally, we conducted stratified analyses across clinically relevant subgroups and performed comprehensive sensitivity analyses to evaluate the robustness of our findings.

**Results:**

A total of 6,173 participants were included in our study. Two depressive-symptom trajectories were identified based on the GBTM analysis. After adjusting for potential covariates, education level was inversely associated with depressive symptoms. Specifically, Individuals with middle school, high school, and college and above education had ORs of 0.70 (95% CI: 0.61–0.80, *P* < 0.001), 0.62 (95% CI: 0.51–0.75, *P* < 0.001), and 0.56 (95% CI: 0.34–0.95, *P* = 0.030), respectively, compared to those with an elementary school or below education. Moreover, stratified analyses across predefined subgroups and sensitivity analyses under different model specifications confirmed the robustness of the outcomes.

**Conclusions:**

The findings suggest that higher education levels were associated with a more favorable trajectory of depressive symptoms. However, given the observational nature of this study, prospective or Mendelian randomization investigations are warranted to further examine this relationship.

**Supplementary Information:**

The online version contains supplementary material available at 10.1186/s40359-026-04794-x.

## Introduction

Depression, a prevalent mental health issue affecting over 350 million people globally, has a lifetime prevalence of up to 6.8% in adults [1]. In China, 23.6% of elderly people experience depressive symptoms, and this rate is steadily increasing [2]. Depression is characterized by a diverse array of symptoms that significantly impair psychological and social functioning, ultimately reducing overall quality of life [3]. These symptoms include a persistently low mood, loss of appetite, and psychomotor slowing [4]. Additionally, individuals may experience suicidal thoughts, sleep disturbances, and pervasive feelings of guilt, worthlessness, hopelessness, and helplessness [5]. Moreover, depression has been confirmed to increase the risk for cardiovascular diseases, dementia, metabolic diseases, and cancer [6–8]. China, a large country experiencing rapid aging, urgently needs to identify risk factors and mechanisms associated with depressive symptoms to implement timely preventative and interventional strategies.

The etiology of depression is multifactorial, involving biological, psychological, and social factors [9, 10] Previous studies indicated that lower educational attainment was associated with higher rates of depressive symptoms [11, 12]. In a cross-sectional study of Korean adults aged 15–69, individuals with fewer than 6 or 12 years of education showed higher levels of depressive symptoms compared to those with 13 or more years of education [11]. In addition, a meta-analysis found that each additional year of education reduced the log odds of depression by 3%, demonstrating a dose-response relationship between education level and depression [12]. However, previous studies examining the relationship between education level and depressive symptoms have assessed these symptoms only once, failing to track the progression of the condition. The Group-Based Trajectory Model (GBTM) is a finite mixture modeling technique that estimates multiple trajectories simultaneously, addressing the limitations of traditional models that only calculate a mean value for the entire study population [13]. Therefore, we employed GBTM to identify distinct patterns of depressive symptom levels based on depressive symptom scores across five waves of the CHARLS. Subsequently, we conducted multivariable logistic regressions to evaluate the association between education level and depressive symptom trajectories among Chinese middle-aged and elderly adults. We hope this study will provide valuable information on targeted policy formulation and interventions for the mental health of middle-aged and older adults.

## Materials and methods

### Study population

This study utilized data from five waves of the CHARLS: 2011, 2013, 2015, 2018, and 2020. The CHARLS is a national cohort survey that employs a multistage sampling strategy, initiated with the 2011 national baseline survey. This extensive survey encompasses 28 provinces, 150 counties, and 450 villages throughout China. Follow-up surveys were conducted in 2013, 2015, 2018, and 2020, respectively, to gather comprehensive data on the health status and related factors affecting the Chinese middle-aged and older population. The CHARLS received approval from the Biomedical Ethics Committee of Peking University, and all participants gave written informed consent. Individuals under 45, those lost to follow-up from wave 2 to 5, and those with incomplete depressive assessments and education data were excluded from the study.

### Assessment of depressive symptoms

Depressive symptoms across the five waves were assessed using the 10-item Center for Epidemiologic Studies Depression Scale (CESD-10). The CESD-10 is a validated and widely used tool for measuring depressive symptoms in China. Participants reported how often they experienced each of the 10 items, encompassing 2 optimistic and 8 negative states, over the past week. Responses ranged from rarely or never (< 1 day), sometimes (1–2 days), occasionally (3–4 days), to most or all of the time (5–7 days). Each item is scored from 0 (rarely) to 3 (most or all the time). The CESD-10 scores ranged from 0 to 30, with higher scores indicating more severe depressive symptoms. According the previous studies, participants with a total score of 10 or higher were defined as having depressive symptoms [14, 15].

### Assessment of education level

The questionnaire asked participants about their highest level of education, categorized as: elementary school or below, middle school, high school, and college or above.

### Assessment of covariates

Drawing from prior studies and clinical practice, we adjusted several covariates to minimize potential confounding effects. The data for the following covariates were sourced from the 2011 baseline survey of CHARLS. Demographic characteristics comprised gender, age, marital status (married or unmarried), and residence (rural or urban). Socioeconomic characteristics included health insurance (urban employee medical insurance, urban and rural resident medical insurance, other medical insurance, or no insurance), and household consumption. Health-related behaviors included smoking status (current smokers, past smokers, or never smokers), drinking status (drink more than once a month, drink but less than once a month, or do not drink), physical activities (vigorous, moderate, or other), and social activities. Health-related factors included self rated health ( good, fair, or poor), body mass index (BMI), and C-reactive protein (CRP). Chronic diseases included hypertension, dyslipidemia, diabetes, cancer or malignant tumor, chronic lung diseases, liver diseases, heart diseases, stroke, kidney diseases, stomach or other digestive diseases, memory related diseases, arthritis or rheumatism, and asthma.

BMI was calculated as weight divided by height squared. Hypertension was defined as a systolic blood pressure of ≥ 140 mmHg, a diastolic blood pressure of ≥ 90 mmHg, or a self-reported diagnosis by a physician. Dyslipidemia was defined by any of the following criteria: total cholesterol ≥ 238.93 mg/dL, triglycerides ≥ 200.90 mg/dL, low density lipoprotein cholesterol ≥ 159.80 mg/dL, or a self-reported physician diagnosis. Diabetes was defined by a glycosylated hemoglobin level of ≥ 6.5% or a self-reported diagnosis confirmed by a physician. Other chronic diseases were self-reported by respondents.

### Statistical analysis

We employed multiple imputation to mitigate bias and enhance precision and statistical power by addressing missing covariate data. Continuous variables were presented as mean and standard deviation (SD) for normally distributed data, or as median and interquartile range (IQR) for skewed data. Categorical variables were reported as frequencies and percentages. We applied the chi-square test or Fisher’s exact test for categorical variables, one-way ANOVA for normally distributed continuous variables, and the Kruskal-Wallis test for non-normally distributed continuous variables.

GBTM was used to identify participants with similar longitudinal trajectories of depressive symptoms, based on CESD-10 scores from five waves (wave 1 to wave 5). To determine the optimal number of depressive symptom trajectories, models ranging from 2-group to 5-group were fitted. The model that demonstrated the best fit was selected based on several criteria: firstly, it exhibited the minimum absolute values for both the Bayesian Information Criterion (BIC) and the Akaike Information Criterion (AIC); secondly, the average posterior probability (AvePP) for each trajectory group was equal to or greater than 70%; and finally, each trajectory group contained more than 5% of the total number of participants [16, 17].

Multivariable logistic regressions were applied to estimate the odds ratios (ORs) and 95% confidence intervals (CIs) for the association of education level with depressive symptom trajectories. Four models were developed using different combinations of covariates. More specifically, model 1 was unadjusted; model 2 was adjusted for demographic and socioeconomic characteristics; model 3 was further adjusted for health-related behaviors and health-related factors; and model 4 was additionally adjusted for chronic diseases. We performed four sensitivity analyses to assess the robustness of the findings. First, we repeated the primary analyses after excluding participants with missing covariate data. Second, we repeated the primary analyses after excluding those with memory-related diseases at baseline. Third, we conducted an analysis regarding education as a continuous variable. Fourth, we conducted a analysis that included individuals with data from at least three measurements of depressive symptoms. Moreover, stratified analyses were conducted to examine whether gender, age, marital status, and residence modified the relationship between education level and depressive symptom trajectories. Statistical analyses were performed using R (version 4.4.2), with a two-tailed significance threshold of *P* < 0.05.

## Results

### Baseline characteristics of the study participants

**Fig. 1:**
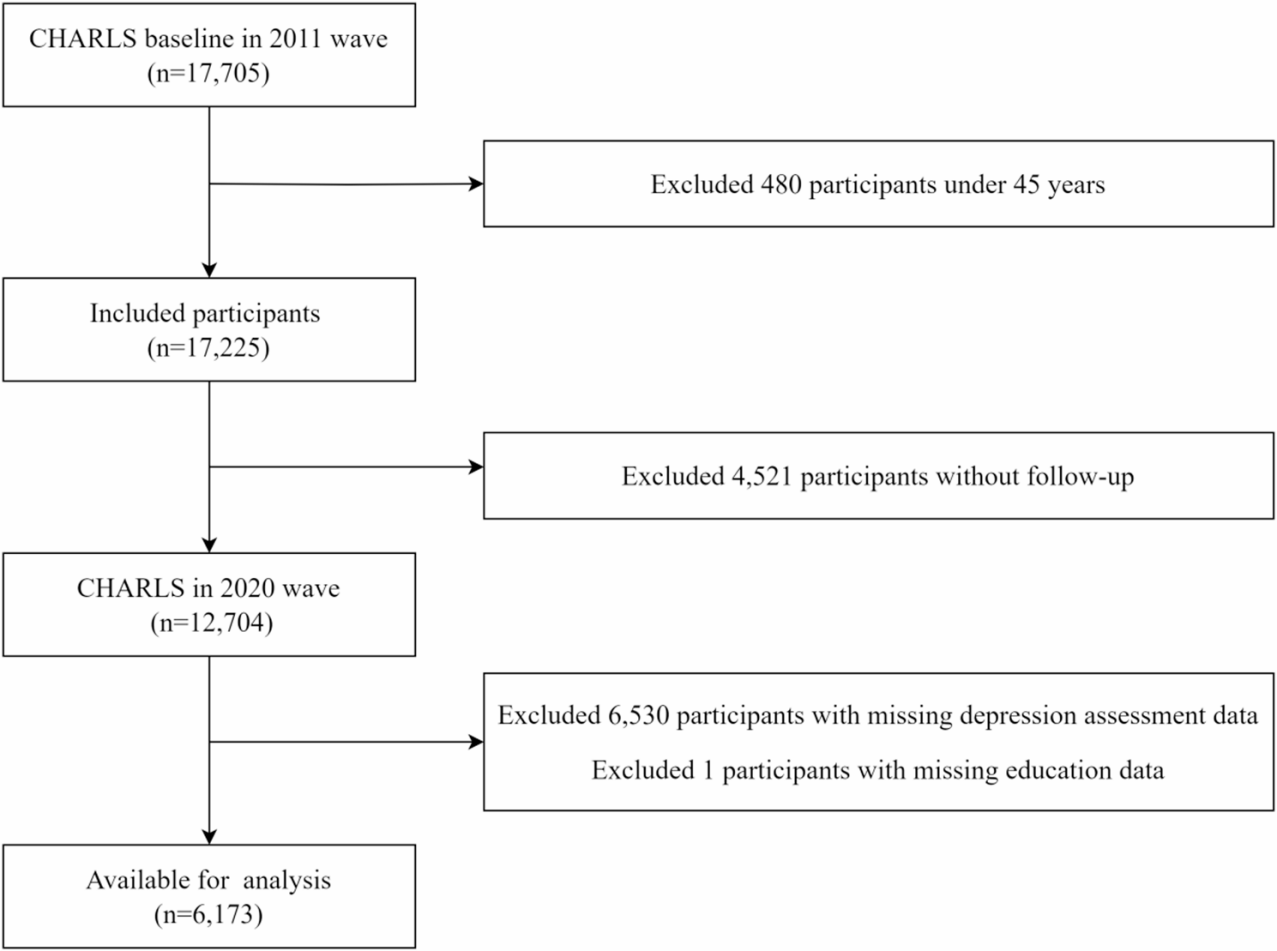
Flow chart of participants selection

A total of 17,705 participants were obtained from the CHARLS database. We excluded 480 participants under 45 years old, 4,521 due to loss to follow-up, 6,530 with missing depression assessment data, and 1 with missing education data. The remaining 6,173 participants were enrolled in our analysis. The exclusion process was illustrated in Fig. 1. Among the included participants, those with higher education levels were predominantly male and tended to exhibit certain socioeconomic and health-related characteristics. Specifically, they reported higher household consumption, were more likely to reside in urban areas, and were frequently married. Additionally, these individuals were often covered by urban employee medical insurance, did not suffer from chronic diseases, and demonstrated healthier lifestyle behaviors, such as never smoking and abstaining from alcohol consumption. They also reported good self-rated health and actively participated in social activities. The baseline characteristics of the included participants were presented in Table 1.

**Table 1:** Baseline characteristics of the included participants

Variables	Total (*n* = 6,173)	Elementary school or below ( *n* = 3,752)	Middle school (*n* = 1,560)	High school (*n* = 745 )	College or above (*n* = 116)	*P*-value
Age (year), Mean ± SD	56.22 ± 7.57	57.68 ± 7.47	53.75 ± 7.06	54.11 ± 6.96	55.94 ± 9.05	< 0.001
CRP (mg/L), Median (IQR)	0.99 (0.54, 1.99)	0.97 (0.53,1.97)	1.02 (0.54,2.00)	1.05 (0.56,2.14)	0.91 (0.55,1.84)	0.745
BMI (kg/m^2^), Mean ± SD	23.88 ± 3.92	23.64 ± 3.91	24.22 ± 3.88	24.30 ± 4.09	24.28 ± 2.84	< 0.001
Household consumption (yuan), Median (IQR)	17,140.00 (94,80.00, 28,340.00)	14,871.00 (83,72.00, 25,310.00)	18,824.00 (10814.00, 30315.00)	22,446.00 (13,256.00, 35,820.00)	36,130.00 (21,460. 00,54,708.00)	< 0.001
Gender, n (%)						< 0.001
Male	3,042 (49.28)	1,549 (41.28)	932 (59.74)	483 (64.83)	78 (67.24)	
Female	3,131 (50.72)	2,203 (58.72)	628 (40.26)	262 (35.17)	38 (32.76)	
Residence, n (%)						< 0.001
Rural	4,987 (80.79)	3,380 (90.09)	1,176 (75.38)	418 (56.11)	13 (11.21)	
Urban	1,186 (19.21)	372 (9.91)	384 (24.62)	327 (43.89)	103 (88.79)	
Marital status, n (%)						< 0.001
Married	5,692 (92.21)	3,398 (90.57)	1,478 (94.74)	706 (94.77)	110 (94.83)	
Not married	481 (7.79)	354 (9.43)	82 (5.26)	39 (5.23)	6 (5.17)	
Health insurance, n (%)						< 0.001
Urban employee medical insurance	672 (10.89)	135 (3.60)	210 (13.46)	233 (31.28)	94 (81.03)	
Urban and rural resident medical insurance	5,152 (83.46)	3,425 (91.28)	1,261 (80.83)	450 (60.40)	16 (13.79)	
Other medical insurance	70 (1.13)	25 (0.67)	19 (1.22)	21 (2.82)	5 (4.31)	
No insurance	279 (4.52)	167 (4.45)	70 (4.49)	41 (5.50)	1 (0.86)	
Hypertension, n (%)						0.042
No	4,042 (65.48)	2,410 (64.23)	1,053 (67.50)	507 (68.05)	72 (62.07)	
Yes	2,131 (34.52)	1,342 (35.77)	507 (32.50)	238 (31.95)	44 (37.93)	
Dyslipidemia, n (%)						0.002
No	4,597 (74.47)	2,820 (75.16)	1,172 (75.13)	534 (71.68)	71 (61.21)	
Yes	1,576 (25.53)	932 (24.84)	388 (24.87)	211 (28.32)	45 (38.79)	
Diabetes, n (%)						0.008
No	5,760 (93.31)	3,519 (93.79)	1,446 (92.69)	695 (93.29)	100 (86.21)	
Yes	413 (6.69)	233 (6.21)	114 (7.31)	50 (6.71)	16 (13.79)	
Cancer or malignant tumor, n (%)						0.667
No	6,126 (99.24)	3,722 (99.20)	1,547 (99.17)	741 (99.46)	116 (100.00)	
Yes	47 (0.76)	30 (0.80)	13 (0.83)	4 (0.54)	0 (0.00)	
Chronic lung diseases, n (%)						< 0.001
No	5,628 (91.17)	3,373 (89.90)	1,461 (93.65)	683 (91.68)	111 (95.69)	
Yes	545 (8.83)	379 (10.10)	99 (6.35)	62 (8.32)	5 (4.31)	
Liver diseases, n (%)						0.099
No	5,914 (95.80)	3,602 (96.00)	1,491 (95.58)	715 (95.97)	106 (91.38)	
Yes	259 (4.20)	150 (4.00)	69 (4.42)	30 (4.03)	10 (8.62)	
Heart diseases, n (%)						< 0.001
No	5,476 (88.71)	3,321 (88.51)	1,407 (90.19)	658 (88.32)	90 (77.59)	
Yes	697 (11.29)	431 (11.49)	153 (9.81)	87 (11.68)	26 (22.41)	
Stroke, n (%)						0.420
No	6,083 (98.54)	3,694 (98.45)	1,543 (98.91)	733 (98.39)	113 (97.41)	
Yes	90 (1.46)	58 (1.55)	17 (1.09)	12 (1.61)	3 (2.59)	
Kidney diseases, n (%)						0.443
No	5,796 (93.89)	3,511 (93.58)	1,470 (94.23)	703 (94.36)	112 (96.55)	
Yes	377 (6.11)	241 (6.42)	90 (5.77)	42 (5.64)	4 (3.45)	
Stomach or other digestive diseases, n (%)						< 0.001
No	4,744 (76.85)	2,782 (74.15)	1,265 (81.09)	605 (81.21)	92 (79.31)	
Yes	1,429 (23.15)	970 (25.85)	295 (18.91)	140 (18.79)	24 (20.69)	
Memory related diseases, n (%)						0.678
No	6,126 (99.24)	3,724 (99.25)	1,549 (99.29)	739 (99.19)	114 (98.28)	
Yes	47 (0.76)	28 (0.75)	11 (0.71)	6 (0.81)	2 (1.72)	
Arthritis or rheumatism, n (%)						< 0.001
No	4,126 (66.84)	2,326 (61.99)	1,145 (73.40)	562 (75.44)	93 (80.17)	
Yes	2,047 (33.16)	1,426 (38.01)	415 (26.60)	183 (24.56)	23 (19.83)	
Asthma, n (%)						0.111
No	5,996 (97.13)	3,629 (96.72)	1,527 (97.88)	727 (97.58)	113 (97.41)	
Yes	177 (2.87)	123 (3.28)	33 (2.12)	18 (2.42)	3 (2.59)	
Physical activities, n (%)						< 0.001
Vigorous activities	2,541 (41.16)	1,644 (43.82)	631 (40.45)	246 (33.02)	20 (17.24)	
Moderate activities	1,851 (29.99)	1,098 (29.26)	446 (28.59)	254 (34.09)	53 (45.69)	
Other activities	1,781 (28.85)	1,010 (26.72)	483 (30.96)	245 (32.89)	43 (37.07)	
Social activities, n (%)						< 0.001
No	2,972 (48.15)	1,960 (52.24)	716 (45.90)	277 (37.18)	19 (16.38)	
Yes	3,201 (51.85)	1,792 (47.76)	844 (54.10)	468 (62.82)	97 (83.62)	
Smoking status, n (%)						< 0.001
Current smokers	1,949 (31.57)	1,081 (28.81)	569 (36.47)	274 (36.78)	25 (21.55)	
Past smokers	500 (8.10)	275 (7.33)	130 (8.33)	79 (10.60)	16 (13.79)	
Never smokers	3,724 (60.33)	2,396 (63.86)	861 (55.19)	392 (52.62)	75 (64.66)	
Drinking status, n (%)						< 0.001
Drink more than once a month	1,645 (26.65)	873 (23.27)	486 (31.15)	240 (32.21)	46 (39.66)	
Drink but less than once a month	504 (8.16)	270 (7.20)	133 (8.53)	87 (11.68)	14 (12.07)	
Do not drink	4,024 (65.19)	2,609 (69.54)	941 (60.32)	418 (56.11)	56 (48.28)	
Self rated health, n (%)						< 0.001
Good	1,599 (25.90)	868 (23.13)	457 (29.29)	231 (31.01)	43 (37.07)	
Fair	3,047 (49.36)	1,807 (48.16)	792 (50.77)	393 (52.75)	55 (47.41)	
Poor	1,527 (24.74)	1,077 (28.70)	311 (19.94)	121 (16.24)	18 (15.52)	

*CRP* C-reactive protein, *BMI* body mass index, *SD* standard deviation, *IQR* interquartile range

### Trajectories of depressive symptoms

**Fig. 2:**
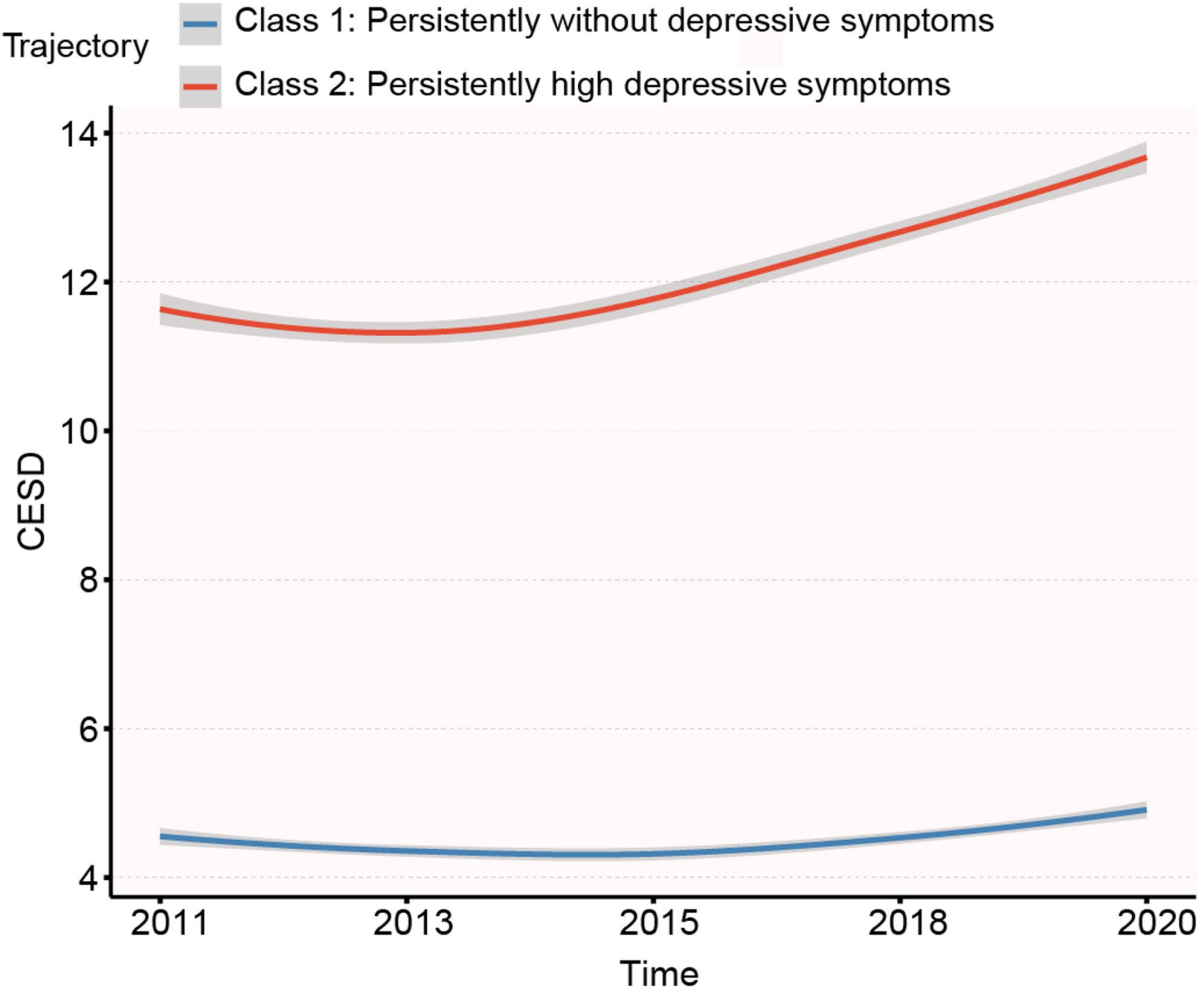
Trajectories of depressive symptoms

Using GBTM, we determined the optimal number of trajectories to account for the variability in depressive symptoms scores from wave 1 to wave 5. Although higher-order trajectory models (three to five classes) were associated with further improvements in the BIC and AIC, these models produced several trajectories with an AvePP below 0.70 (Table 2). Specifically, in the three-trajectory model, one class (Class 3) had an AvePP of 0.68. In the four-trajectory model, two classes exhibited low AvePP: Class 2 (0.65) and Class 4 (0.60). In the five-trajectory model, three classes showed inadequate AvePP values: Class 2 (0.51), Class 4 (0.56), and Class 5 (0.55). Therefore, the GBTM model with two trajectories was deemed optimal. Figure 2 illustrates two longitudinal trajectories of depressive symptom scores over the survey years: persistently without depressive symptoms (*N* = 3,271, 52.99%) and persistently high depressive symptoms (*N* = 2,902, 47.01%). The baseline characteristics of the participants in each depressive symptom trajectory group were presented in Supplementary Table 1. Participants in the persistently high depressive symptoms trajectory group were more likely to be female, reside in rural areas, have lower levels of education and household consumption, not married, lack health insurance, suffer from chronic diseases, report poor self rated health, and refrain from engaging in social activities compared to those in the persistently without depressive symptom trajectory group.

**Table 2:** Fit statistics of the different subgroup depressive-symptom trajectory models

Group	AIC	BIC	Class proportion (%)					AvePP				
			Class 1	Class 2	Class 3	Class 4	Class 5	Class 1	Class 2	Class 3	Class 4	Class 5
2	187,385.99	187,486.91	52.99	47.01	NA	NA	NA	0.86	0.91	NA	NA	NA
3	187,008.65	187,143.20	39.46	28.03	32.51	NA	NA	0.84	0.87	0.68	NA	NA
4	186,954.03	187,122.23	38.90	29.40	25.85	5.85	NA	0.83	0.65	0.83	0.60	NA
5	186,922.48	187,124.32	36.71	19.25	19.73	5.67	18.65	0.81	0.51	0.81	0.56	0.55

*NA* Not available, *AIC* Akaike Information Criterion, *BIC* Bayesian Information Criterion, *AvePP* average posterior probability

### Association between education level and depressive symptom trajectories

Multivariable logistic regression analyses revealed a negative association between education level and persistently high depressive symptom trajectory, after adjusting for all potential covariates (Table 3). Compared with individuals with elementary school or below, the adjusted OR values for middle school, high school, and college or above were 0.70 (95% CI: 0.61–0.80, *P* < 0.001), 0.62 (95% CI: 0.51–0.75, *P* < 0.001), and 0.56 (95% CI: 0.34–0.95, *P* = 0.030), respectively. In addition, the inverse associations were consistent across models with various covariate adjustments.

**Table 3:** Association between education level and depressive symptom trajectories

Education level	Event (%)	Model 1		Model 2		Model 3		Model 4	
		OR (95%CI)	*P*-value	OR (95%CI)	*P*-value	OR (95%CI)	*P*-value	OR (95%CI)	*P*-value
Elementary school or below	2,061 (54.93)	1 (Ref)		1 (Ref)		1 (Ref)		1 (Ref)	
Middle school	589 (37.76)	0.50 (0.44–0.56)	< 0.001	0.62 (0.54–0.71)	< 0.001	0.67 (0.58–0.77)	< 0.001	0.70 (0.61–0.80)	< 0.001
High school	229 (30.74)	0.36 (0.31–0.43)	< 0.001	0.54 (0.45–0.65)	< 0.001	0.60 (0.49–0.73)	< 0.001	0.62 (0.51–0.75)	< 0.001
College or above	23 (19.83)	0.20 (0.13–0.32)	< 0.001	0.45 (0.27–0.73)	0.001	0.54 (0.32–0.90)	0.018	0.56 (0.34–0.95)	0.030
Trend test			< 0.001		< 0.001		< 0.001		< 0.001

*CI* confidence interval, *OR* odds ratio, *model 1* was unadjusted, *model 2* was adjusted for demographic and socioeconomic characteristics, *model 3* was further adjusted for health-related behaviors and health-related factors, and *model 4* was additionally adjusted for chronic diseases

### Stratified and sensitivity analyses

**Fig. 3:**
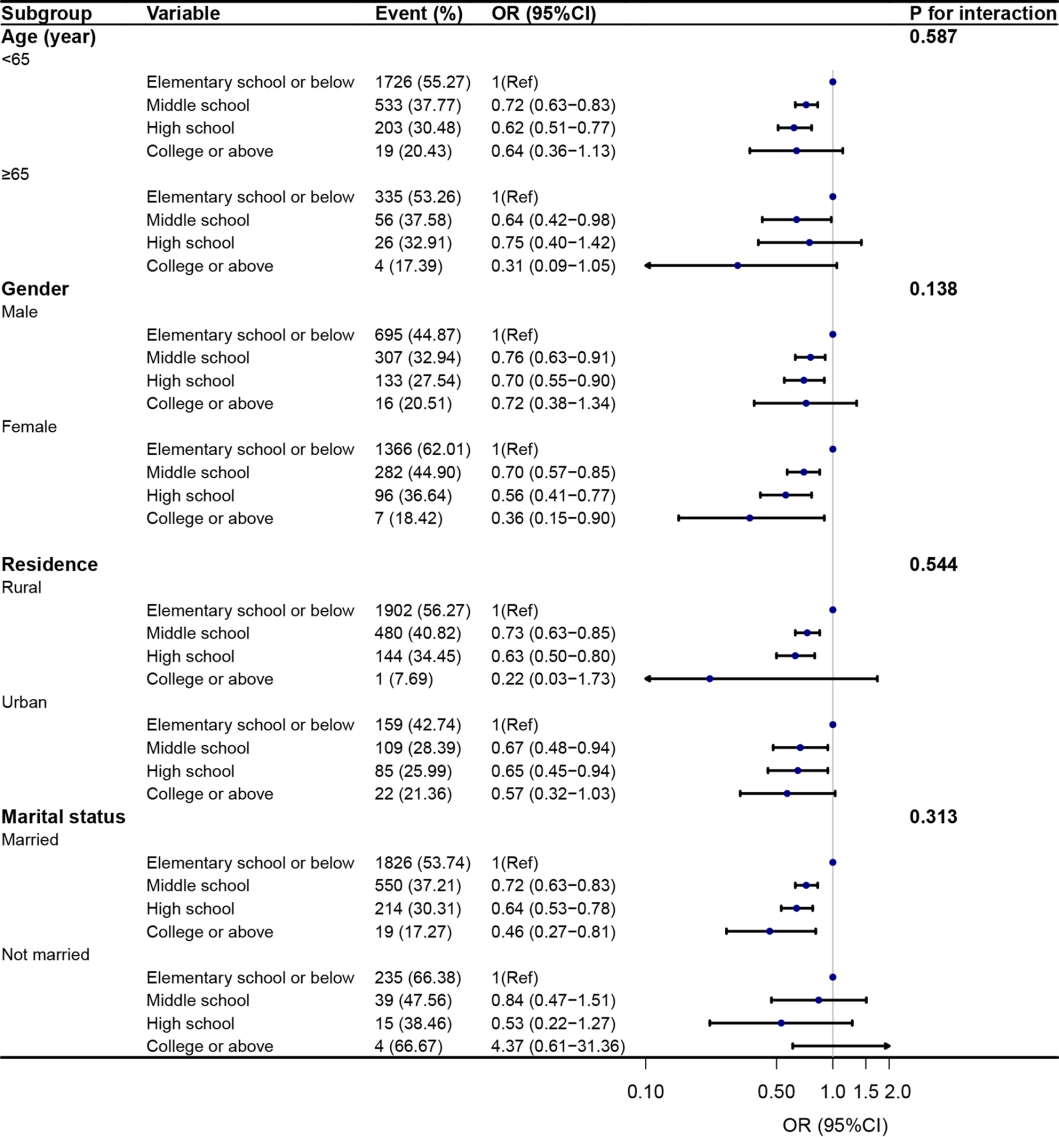
Stratified analyses of the association between education level and depressive symptom trajectories. Stratifications were adjusted for demographic and socioeconomic characteristics, health-related behaviors and factors, and chronic diseases, excluding the specific stratification component

Stratified analyses revealed a consistent association between education level and depressive symptom trajectories across various subgroups (Fig. 3). Additionally, no interactions between education level and these subgroup variables were observed in relation to depressive symptom trajectories (*P* > 0.05). The results remained robust after excluding individuals with incomplete covariate data (Supplementary Table 2). The adjusted ORs showed a decreasing trend relative to the reference category (elementary school or below): 0.67 (95% CI: 0.56–0.79, *P* < 0.001) for middle school, 0.55 (95% CI: 0.43–0.71, *P* < 0.001) for high school, and 0.48 (95% CI: 0.23–0.99, *P* = 0.048) for college or above. Similarly, the results remained robust after excluding individuals with memory-related diseases at baseline (Supplementary Table 3). The adjusted ORs again demonstrated a decreasing trend compared with elementary school or below: 0.70 (95% CI: 0.61–0.81, *P* < 0.001) for middle school, 0.62 (95% CI: 0.51–0.76, *P* < 0.001) for high school, and 0.54 (95% CI: 0.32–0.91, *P* = 0.021) for college or above. When education was treated as a continuous variable, a consistent inverse association was observed between educational level and a persistently high trajectory of depressive symptoms (OR = 0.77, 95% CI: 0.71–0.84, *P* < 0.001) (Supplementary Table 4). Furthermore, the results remained robust when including individuals with data from at least three measurements of depressive symptoms (Supplementary Table 5). The depressive symptom trajectories derived from three measurements of depressive symptoms data (*n* = 12,754) were similar to those from the main analyses: a “without depressive symptoms” trajectory (*n* = 6,722, 52.71%) and a “persistently high depressive symptoms” trajectory (*n* = 6,032, 47.29%). The adjusted ORs showed a decreasing trend relative to the reference category (elementary school or below): 0.73 (95% CI: 0.66–0.80, *P* < 0.001) for middle school, 0.60 (95% CI: 0.52–0.69, *P* < 0.001) for high school, and 0.48 (95% CI: 0.33–0.69, *P* < 0.001) for college or above.

## Discussion

In this large nationally representative cohort analysis, we investigated the relationship between education level and long-term depressive symptom trajectories in middle-aged and older Chinese individuals. Two depressive symptom trajectories were identified: persistently without depressive symptoms and persistently high depressive symptoms. We found that higher education levels were positively associated with more favorable trajectories of depressive symptoms.

Our study employed GBTM to identify two depressive symptom trajectories, which differs from the three or more trajectory levels commonly reported in the existing literature [18–22]. This discrepancy in the number of trajectories likely reflects heterogeneity across studies in participant inclusion and exclusion criteria, the number of depressive symptom assessments, and follow-up durations [18–22]. Unlike some previous studies that excluded participants with depressive symptoms at baseline [18–20], our study did not apply such exclusion. Furthermore, we included only participants with complete depressive symptom assessments across all five waves, whereas other studies often included those with two or more waves of assessments [18–20, 22]. Lastly, we determined the optimal number of trajectories based on variability in depressive symptom scores from wave 1 to wave 5, while other studies typically used follow-up periods from wave 1 to wave 4 or wave 1 to wave 3 [18, 21].

Our findings align with previous research indicating that education level was inversely associated with depressive symptoms [23–25]. A cross-sectional survey of 4,945 older adults from a nationwide representative Chinese sample found a negative association between education level and depression [23]. Additionally, in a cross-sectional analysis based on 22 European countries, Individuals with lower educational attainment, specifically lower secondary education or less, generally exhibited a higher risk of depressive symptoms [24]. However, this association showed significant cross-national variation, with strong associations in Hungary and Slovenia, but not in Austria, Denmark, and Estonia [24]. Moreover, a study utilizing data from the Survey of Health, Ageing and Retirement in Europe (SHARE) found that couples over 50 with low education levels related a higher depressive symptoms (OR = 1.64, 95%CI: 1.07–2.52) [25]. Nevertheless, the depressive symptoms in these studies were typically measured only once. This methodology may lack precision in assessing depressive symptoms given that manifestations of depressive symptoms typically demonstrate dynamic variability across the lifespan [26]. Thus, we analyzed the progression of depressive symptoms, instead of a single measurement, to identify clear relationships between education level and depressive symptoms. This study provides new evidence supporting the inverse association of education level with long-term depressive symptoms, highlighting the need to focus on populations with low education levels and, where necessary, to develop corresponding measures to reduce the occurrence of depressive symptoms. Furthermore, improving the education level of the population might help reduce depressive symptoms.

Multiple neuropsychological pathways potentially mediate the association between educational attainment and depressive symptoms [27–29]. Primarily, educational achievement might confer psychological resilience through enhanced socioeconomic resource acquisition [27]. Substantial empirical evidence indicates that advanced education correlates with improved occupational opportunities, resulting in elevated income brackets and superior social positioning [28]. These socioeconomic advantages collectively reduce environmental stressors while increasing life satisfaction metrics, thereby diminishing the risk of depressive symptoms [29]. A mediation analysis utilizing data from the 2018 wave of CHARLS revealed that economic status, health behaviors, and cognitive function significantly mediated this association [30]. Secondarily, formal education cultivates cognitive-emotional competencies that directly buffer against depressive symptoms [31]. Neurocognitive research demonstrates that educational training enhances executive function, emotional regulation capacity, and systematic problem-solving skills [32]. In addition, several studies indicate that college-educated individuals are more likely to maintain healthy lifestyle habits [33]. Furthermore, the educational environment fosters the growth of social capital [32]. Finally, these neurobehavioral and psychosocial mechanisms might together decrease the occurrence of depressive symptoms.

This study has several strengths. First, this study used a large national sample with an extended follow-up period to examine the relationship between education level and depressive symptom trajectories in middle-aged and older Chinese adults, ensuring representative and credible results. Second, given the dynamic and intermittent nature of depressive symptoms, relying solely on a single measure or a cross-sectional analysis is inadequate for uncovering the true correlation between education level and depressive symptoms [34]. To address this limitation, we employed the GBTM approach to track the progression of depressive symptom trajectories over time. This method offers more precise insights into the temporal changes in depressive symptoms compared to assessments conducted at a single time point, thereby enhancing the value of our findings. Third, we accounted for multiple potential confounders to reduce their impact on the results. However, our study has several limitations that must be considered. First, recall bias was unavoidable due to the study’s observational design and dependence on a questionnaire. Second, depressive symptoms were identified using the CESD-10, potentially introducing information bias. However, the CESD-10 demonstrates strong validity and reliability in detecting depressive symptoms, with a Cronbach’s α coefficient of 0.78–0.79 [35]. Third, although the associations remained robust after adjusting for potential confounding factors, the possibility of residual confounding factors cannot be ruled out. Fourth, the observational design permits the identification of associations but does not allow for causal inferences between education level and depressive symptoms. Therefore, prospective studies are warranted to further examine this relationship. Fifth, the covariates were derived from the 2011 baseline survey of CHARLS, which may not fully account for time-varying influences on depressive symptom trajectories. Nevertheless, the primary exposure of interest in this study—baseline education level—is a time-invariant characteristic. Therefore, using baseline measurement for this key independent variable is both necessary and appropriate. Moreover, measuring covariates only at baseline, rather than as time-varying variables, is common practice in many studies [26, 36]. Sixth, the identification of only two trajectory groups (persistently high versus low) substantially reduces the granularity of longitudinal patterns and may underutilize the potential of GBTM. However, based on the model selection criteria for GBTM, the two-group solution was determined to be optimal. Furthermore, several studies have similarly identified two-group depressive symptom trajectories [37, 38] For example, one study employed GBTM to delineate depressive symptom trajectories among stroke survivor–caregiver dyads, identifying two distinct caregiver trajectories: a low-stable group (71.5%) and a high-decreasing group (28.5%) [37]. In another post hoc analysis, a two-class GBTM solution was deemed optimal for classifying depressive symptoms, distinguishing a high/rapidly declining trajectory (39.4%) from a moderate/gradually declining trajectory (60.6%) [38]. Finally, as our participants were from China, future research should assess the applicability of these findings in other populations. Despite its limitations, this large-scale, nationally representative longitudinal survey further supports the role of education level in depressive symptoms.

## Conclusions

This study identified two different trajectories of depressive symptoms among Chinese middle-aged and older adults. Participants with higher education levels were independently associated with a favorable depressive symptom trajectory over time. The findings further suggest that higher education might associated a lower likelihood of depressive symptoms, offering insights into its broader impact on mental health from a macrosociological perspective. However, given the observational nature of our study, prospective or Mendelian randomization studies are warranted to further investigate this relationship.

## Supplementary Information

Supplementary Material 1.

## Data Availability

The data that support the findings of this study are available from the corresponding author upon reasonable request.

## Abbreviations

CHARLS: China Health and Retirement Longitudinal Study
CESD-10: 10-item Center for Epidemiologic Studies Depression Scale
GBTM: Group-based trajectory modeling
BMI: Body mass index
CRP: C-reactive protein
SD: Standard deviation
IQR: Interquartile range
BIC: Bayesian Information Criterion
AIC: Akaike Information Criterion
AvePP: Average posterior probability
OR: Odds ratio
CI: Confidence interval
SHARE: Survey of Health, Ageing and Retirement in Europe
