# Supplementary material for: Association between education level and depressive symptom trajectories in middle-aged and older Chinese adults: a nationally representative cohort study
Source: BMC Psychol. 2026 May 19;14:1031. doi: 10.1186/s40359-026-04794-x (PMC13352782; doi:10.1186/s40359-026-04794-x)
Supplement: Supplementary file 1 — Supplementary Material 1. [file 40359_2026_4794_MOESM1_ESM.docx]

**Supplementary Table 1**. Baseline characteristics of the participants in each depressive symptom trajectory group

| Variables | Total (n = 6173) | Persistently without depressive symptoms (n = 3271) | Persistently high depressive symptoms (n = 2902) | *P*-value |
| --- | --- | --- | --- | --- |
|  |  |  |  |  |
| Age (year), Mean ± SD | 56.22 ± 7.57 | 56.07 ± 7.69 | 56.40 ± 7.42 | 0.086 |
| CRP (mg/L), Median (IQR) | 0.99 (0.54, 1.99) | 1.01 (0.55, 1.94) | 0.97 (0.53, 2.04) | 0.449 |
| BMI (kg/m^2^), Mean ± SD | 23.88 ± 3.92 | 24.09 ± 3.89 | 23.63 ± 3.94 | <0.001 |
| Household consumption (yuan), Median (IQR) | 17,140.00 (9,480.00, 28,340.00) | 18,520.00 (9,992.00, 30,480.00) | 15,710.00 (8,845.00, 25,918.75) | <0.001 |
| Gender, n (%) |  |  |  | <0.001 |
| Male | 3,042 (49.28) | 1,891 (57.81) | 1,151 (39.66) |  |
| Female | 3,131 (50.72) | 1,380 (42.19) | 1,751 (60.34) |  |
| Residence, n (%) |  |  |  | <0.001 |
| Rural | 4,987 (80.79) | 2,460 (75.21) | 2,527 (87.08) |  |
| Urban | 1,186 (19.21) | 811 (24.79) | 375 (12.92) |  |
| Education level, n(%) |  |  |  | <0.001 |
| Elementary school or below | 3,752 (60.78) | 1,691 (51.70) | 2,061 (71.02) |  |
| Middle school | 1,560 (25.27) | 971 (29.69) | 589 (20.30) |  |
| High school | 745 (12.07) | 516 (15.77) | 229 (7.89) |  |
| College or above | 116 (1.88) | 93 (2.84) | 23 (0.79) |  |
| Marital status, n (%) |  |  |  | <0.001 |
| Married | 5,692 (92.21) | 3,083 (94.25) | 2,609 (89.90) |  |
| Not married | 481 (7.79) | 188 (5.75) | 293 (10.10) |  |
| Health insurance, n (%) |  |  |  | <0.001 |
| Urban employee medical insurance | 672 (10.89) | 497 (15.19) | 175 (6.03) |  |
| Urban and rural resident medical insurance | 5,152 (83.46) | 2,580 (78.87) | 2,572 (88.63) |  |
| Other medical insurance | 70 (1.13) | 49 (1.50) | 21 (0.72) |  |
| No insurance | 279 (4.52) | 145 (4.43) | 134 (4.62) |  |
| Hypertension, n (%) |  |  |  | 0.548 |
| No | 4,042 (65.48) | 2,153 (65.82) | 1,889 (65.09) |  |
| Yes | 2,131 (34.52) | 1,118 (34.18) | 1,013 (34.91) |  |
| Dyslipidemia, n (%) |  |  |  | 0.353 |
| No | 4,597 (74.47) | 2,420 (73.98) | 2,177 (75.02) |  |
| Yes | 1,576 (25.53) | 851 (26.02) | 725 (24.98) |  |
| Diabetes, n (%) |  |  |  | 0.621 |
| No | 5,760 (93.31) | 3,057 (93.46) | 2,703 (93.14) |  |
| Yes | 413 (6.69) | 214 (6.54) | 199 (6.86) |  |
| Cancer or malignant tumor, n (%) |  |  |  | 0.748 |
| No | 6,126 (99.24) | 3,245 (99.21) | 2,881 (99.28) |  |
| Yes | 47 (0.76) | 26 (0.79) | 21 (0.72) |  |
| Chronic lung diseases, n (%) |  |  |  | <0.001 |
| No | 5,628 (91.17) | 3,064 (93.67) | 2,564 (88.35) |  |
| Yes | 545 (8.83) | 207 (6.33) | 338 (11.65) |  |
| Liver diseases, n (%) |  |  |  | 0.010 |
| No | 5,914 (95.80) | 3,154 (96.42) | 2,760 (95.11) |  |
| Yes | 259 (4.20) | 117 (3.58) | 142 (4.89) |  |
| Heart diseases, n (%) |  |  |  | <0.001 |
| No | 5476 (88.71) | 2970 (90.80) | 2506 (86.35) |  |
| Yes | 697 (11.29) | 301 (9.20) | 396 (13.65) |  |
| Stroke, n (%) |  |  |  | <0.001 |
| No | 6,083 (98.54) | 3,239 (99.02) | 2,844 (98.00) |  |
| Yes | 90 (1.46) | 32 (0.98) | 58 (2.00) |  |
| Kidney diseases, n (%) |  |  |  | <0.001 |
| No | 5,796 (93.89) | 3,131 (95.72) | 2,665 (91.83) |  |
| Yes | 377 (6.11) | 140 (4.28) | 237 (8.17) |  |
| Stomach or other digestive diseases, n (%) |  |  |  | <0.001 |
| No | 4,744 (76.85) | 2,699 (82.51) | 2,045 (70.47) |  |
| Yes | 1,429 (23.15) | 572 (17.49) | 857 (29.53) |  |
| Memory related diseases, n (%) |  |  |  | 0.043 |
| No | 6,126 (99.24) | 3,253 (99.45) | 2,873 (99.00) |  |
| Yes | 47 (0.76) | 18 (0.55) | 29 (1.00) |  |
| Arthritis or rheumatism, n (%) |  |  |  | <0.001 |
| No | 4,126 (66.84) | 2,463 (75.30) | 1,663 (57.31) |  |
| Yes | 2047 (33.16) | 808 (24.70) | 1,239 (42.69) |  |
| Asthma, n (%) |  |  |  | <0.001 |
| No | 5,996 (97.13) | 3,209 (98.10) | 2,787 (96.04) |  |
| Yes | 177 (2.87) | 62 (1.90) | 115 (3.96) |  |
| Physical activities, n (%) |  |  |  | <0.001 |
| Vigorous activities | 2,541 (41.16) | 1,285 (39.28) | 1,256 (43.28) |  |
| Moderate activities | 1,851 (29.99) | 1,002 (30.63) | 849 (29.26) |  |
| Other activities | 1,781 (28.85) | 984 30.08) | 797 (27.46) |  |
| Social activities, n (%) |  |  |  | <0.001 |
| No | 2,972 (48.15) | 1,433 (43.81) | 1,539 (53.03) |  |
| Yes | 3,201 (51.85) | 1,838 (56.19) | 1,363 (46.97) |  |
| Smoking status, n (%) |  |  |  | <0.001 |
| Current smokers | 1,949 (31.57) | 1,143 (34.94) | 806 (27.77) |  |
| Past smokers | 500 (8.10) | 293 (8.96) | 207 (7.13) |  |
| Never smokers | 3,724 (60.33) | 1,835 (56.10) | 1,889 (65.09) |  |
| Drinking status, n (%) |  |  |  | <0.001 |
| Drink more than once a month | 1,645 (26.65) | 1,023 (31.27) | 622 (21.43) |  |
| Drink but less than once a month | 504 (8.16) | 289 (8.84) | 215 (7.41) |  |
| Do not drink | 4,024 (65.19) | 1,959 (59.89) | 2,065 (71.16) |  |
| Self rated health, n (%) |  |  |  | <0.001 |
| Good | 1,599 (25.90) | 1,141 (34.88) | 458 (15.78) |  |
| Fair | 3,047 (49.36) | 1,699 (51.94) | 1,348 (46.45) |  |
| Poor | 1,527 (24.74) | 431 (13.18) | 1,096 (37.77) |  |

*Note:* CRP, C-reactive protein; BMI, body mass index; SD, standard deviation; IQR, interquartile range.

**Supplementary Table 2**. Association between education level and depressive symptom trajectories after excluding individuals with missing covariate data

| Education level | Event (%) | Model 1 | | Model 2 | | Model 3 | | Model 4 | |
| --- | --- | --- | --- | --- | --- | --- | --- | --- | --- |
|  |  | OR (95%CI) | *P*-value | OR (95%CI) | *P*-value | OR (95%CI) | *P*-value | OR (95%CI) | *P*-value |
| Elementary school or below | 1,404 (56.16) | 1 (Ref) |  | 1 (Ref) |  | 1 (Ref) |  | 1 (Ref) |  |
| Middle school | 382 (38.31) | 0.48 (0.42-0.56) | <0.001 | 0.60 (0.51-0.70) | <0.001 | 0.64 (0.54-0.76) | <0.001 | 0.67 (0.56-0.79) | <0.001 |
| High school | 126 (29.58) | 0.33 (0.26-0.41) | <0.001 | 0.47 (0.37-0.60) | <0.001 | 0.54 (0.42-0.69) | <0.001 | 0.55 (0.43-0.71) | <0.001 |
| College or above | 11 (18.64) | 0.18 (0.09-0.35) | <0.001 | 0.43 (0.21-0.87) | 0.019 | 0.45 (0.22-0.92) | 0.029 | 0.48 (0.23-0.99) | 0.048 |
| Trend test |  |  | <0.001 |  | <0.001 |  | <0.001 |  | <0.001 |

*Note*: CI, confidence interval; OR, odds ratio; model 1 was unadjusted; model 2 was adjusted for demographic and socioeconomic characteristics; model 3 was further adjusted for health-related behaviors and health-related factors; and model 4 was additionally adjusted for chronic diseases.

**Supplementary Table 3**. Association between education level and depressive symptom trajectories after excluding memory-related disease at baseline.

| Education level | Event (%) | Model 1 | | Model 2 | | Model 3 | | Model 4 | |
| --- | --- | --- | --- | --- | --- | --- | --- | --- | --- |
|  |  | OR (95%CI) | *P*-value | OR (95%CI) | *P*-value | OR (95%CI) | *P*-value | OR (95%CI) | *P*-value |
| Elementary school or below | 2,042 (54.83) | 1 (Ref) |  | 1 (Ref) |  | 1 (Ref) |  | 1 (Ref) |  |
| Middle school | 582 (37.57) | 0.50 (0.44-0.56) | <0.001 | 0.62 (0.54-0.70) | <0.001 | 0.67 (0.59-0.77) | <0.001 | 0.70 (0.61-0.81) | <0.001 |
| High school | 227 (30.72) | 0.37 (0.31-0.43) | <0.001 | 0.54 (0.45-0.65) | <0.001 | 0.61 (0.50-0.74) | <0.001 | 0.62 (0.51-0.76) | <0.001 |
| College or above | 22 (19.30) | 0.20 (0.12-0.32) | <0.001 | 0.43 (0.26-0.71) | 0.001 | 0.51 (0.30-0.86) | 0.011 | 0.54 (0.32-0.91) | 0.021 |
| Trend test |  | 0.57 (0.53-0.61) | <0.001 | 0.71 (0.65-0.77) | <0.001 | 0.76 (0.69-0.82) | <0.001 | 0.77 (0.71-0.84) | <0.001 |

*Note*: CI, confidence interval; OR, odds ratio; model 1 was unadjusted; model 2 was adjusted for demographic and socioeconomic characteristics; model 3 was further adjusted for health-related behaviors and health-related factors; and model 4 was additionally adjusted for chronic diseases.

**Supplementary Table 4**. Association between education level and depressive symptom trajectories regarding education as a continuous variable.

| Education level | Event (%) | Model 1 | | Model 2 | | Model 3 | | Model 4 | |
| --- | --- | --- | --- | --- | --- | --- | --- | --- | --- |
|  |  | OR (95%CI) | *P*-value | OR (95%CI) | *P*-value | OR (95%CI) | *P*-value | OR (95%CI) | *P*-value |
|  | 2,902 (47.01) | 0.57 (0.53-0.62) | <0.001 | 0.71 (0.65-0.77) | <0.001 | 0.76 (0.70-0.82) | <0.001 | 0.77 (0.71-0.84) | <0.001 |

*Note*: CI, confidence interval; OR, odds ratio; model 1 was unadjusted; model 2 was adjusted for demographic and socioeconomic characteristics; model 3 was further adjusted for health-related behaviors and health-related factors; and model 4 was additionally adjusted for chronic diseases.

**Supplementary Table 5**. Association between education level and depressive symptom trajectories after including individuals with at least three measurements of depressive symptoms.

| Education level | Event (%) | Model 1 | | Model 2 | | Model 3 | | Model 4 | |
| --- | --- | --- | --- | --- | --- | --- | --- | --- | --- |
|  |  | OR (95%CI) | *P*-value | OR (95%CI) | *P*-value | OR (95%CI) | *P*-value | OR (95%CI) | *P*-value |
| Elementary school or below | 4,521 (54.53) | 1 (Ref) |  | 1 (Ref) |  | 1 (Ref) |  | 1 (Ref) |  |
| Middle school | 1,076 (37.83) | 0.51 (0.47-0.55) | <0.001 | 0.65 (0.59-0.72) | <0.001 | 0.71 (0.64-0.78) | <0.001 | 0.73 (0.66-0.80) | <0.001 |
| High school | 392 (28.80) | 0.34 (0.30-0.38) | <0.001 | 0.51 (0.44-0.59) | <0.001 | 0.59 (0.51-0.68) | <0.001 | 0.60 (0.52-0.69) | <0.001 |
| College or above | 43 (16.67) | 0.17 (0.12-0.23) | <0.001 | 0.38 (0.26-0.53) | <0.001 | 0.48 (0.33-0.69) | <0.001 | 0.48 (0.33-0.69) | <0.001 |
| Trend test |  | 0.56 (0.53-0.58) | <0.001 | 0.70 (0.66-0.74) | <0.001 | 0.75 (0.71-0.80) | <0.001 | 0.76 (0.72-0.81) | <0.001 |

*Note*: CI, confidence interval; OR, odds ratio; model 1 was unadjusted; model 2 was adjusted for demographic and socioeconomic characteristics; model 3 was further adjusted for health-related behaviors and health-related factors; and model 4 was additionally adjusted for chronic diseases.
